# Harnessing the Full Power of Chemometric-Based Analysis of Total Reflection X-ray Fluorescence Spectral Data to Boost the Identification of Seafood Provenance and Fishing Areas

**DOI:** 10.3390/foods11172699

**Published:** 2022-09-04

**Authors:** Bernardo Duarte, Renato Mamede, João Carreiras, Irina A. Duarte, Isabel Caçador, Patrick Reis-Santos, Rita P. Vasconcelos, Carla Gameiro, Pedro Ré, Susanne E. Tanner, Vanessa F. Fonseca

**Affiliations:** 1 MARE—Marine and Environmental Sciences Centre & ARNET—Aquatic Research Infrastructure Network Associated Laboratory, Faculdade de Ciências, Universidade de Lisboa, Campo Grande, 1749-016 Lisboa, Portugal; 2 Departamento de Biologia Vegetal, Faculdade de Ciências, Universidade de Lisboa, Campo Grande, 1749-016 Lisboa, Portugal; 3 Southern Seas Ecology Laboratories, School of Biological Sciences, The University of Adelaide, Adelaide, SA 5005, Australia; 4 IPMA—Instituto Português do Mar e da Atmosfera, Av. Dr. Alfredo Magalhães Ramalho 6, 1495-165 Algés, Portugal; 5 MARE—Marine and Environmental Sciences Centre & ARNET—Aquatic Research Infrastructure Network Associated Laboratory, Laboratório Marítimo da Guia, Faculdade de Ciências, Universidade de Lisboa, Avenida Nossa Senhora do Cabo, 2750-374 Cascais, Portugal; 6 Departamento de Biologia Animal, Faculdade de Ciências, Universidade de Lisboa, Campo Grande, 1749-016 Lisboa, Portugal

**Keywords:** traceability, X-ray spectroscopy, chemometrics

## Abstract

Provenance and traceability are crucial aspects of seafood safety, supporting managers and regulators, and allowing consumers to have clear information about the origin of the seafood products they consume. In the present study, we developed an innovative spectral approach based on total reflection X-ray fluorescence (TXRF) spectroscopy to identify the provenance of seafood and present a case study for five economically relevant marine species harvested in different areas of the Atlantic Portuguese coast: three bony fish—*Merluccius merluccius*, *Scomber colias*, and *Sparus aurata*; one elasmobranch—*Raja clavata*; one cephalopod—*Octopus vulgaris*. Applying a first-order Savitzky–Golay transformation to the TXRF spectra reduced the potential matrix physical effects on the light scattering of the X-ray beam while maintaining the spectral differences inherent to the chemical composition of the samples. Furthermore, a variable importance in projection partial least-squares discriminant analysis (VIP-PLS-DA), with k − 1 components (where k is the number of geographical origins of each seafood species), produced robust high-quality models of classification of samples according to their geographical origin, with several clusters well-evidenced in the dispersion plots of all species. Four of the five species displayed models with an overall classification above 80.0%, whereas the lowest classification accuracy for *S. aurata* was 74.2%. Notably, about 10% of the spectral features that significantly contribute to class differentiation are shared among all species. The results obtained suggest that TXRF spectra can be used for traceability purposes in seafood species (from bony and cartilaginous fishes to cephalopods) and that the presented chemometric approach has an added value for coupling with classic TXRF spectral peak deconvolution and elemental quantification, allowing characterization of the geographical origin of samples, providing a highly accurate and informative dataset in terms of food safety.

## 1. Introduction

The strengthening of legal requirements in food safety in recent decades has led to the development and implementation of geographical origin authentication methodologies that allow consumers to know the origin of the food products they consume [1]. To tackle the growing issue of food fraud, the European Union (EU) published a resolution compelling all member states to develop and adopt tools to increase food traceability and prevent mislabeling [2]. Similar regulations worldwide have led to a growing number of studies focused on the development of elemental and biochemical markers that provide natural tags of the geographical location of the product capture and production, without interference from the producer’s report [1]; the development of genetic approaches have greatly advanced the ability to identify species in seafood products even after food processing [3]. Various reasons underlie mislabeling, including (i) involuntary mislabeling of origin and species, (ii) misidentification of closely related species, (iii) misunderstanding of the common names of species [4], or (iv) deliberate mislabeling of species for direct commercial benefit [5], replacing labels of low-value species with economically relevant species, e.g., farmed catfish (*Pangasius* sp.) identified as wild-caught Atlantic cod (*Gadus morhua*) [6]. Moreover, restrictions on the catches of specific species enforced as management measures can lead to intentional food fraud to obfuscate collection in areas or periods where and/or when catches are forbidden [7]. Seafood is among the most economically valuable key components of the human diet, comprising 16.7% of the animal protein consumption per person on a global scale [8]. Moreover, in recent decades, societal concern to pursue a healthier lifestyle and diet has boosted seafood products’ consumption from 9.9 kg per capita in the 1960s to 19.7 kg per capita in 2013 [8]. With this high value and high demand for seafood products, the risk of food fraud associated with mislabeling is greatly amplified, either unintentionally or with the intent to gain profit from illegal practices [7].

Several techniques have been employed to trace the geographical origin of food products, including elemental analysis [9,10,11,12,13,14], isotope analysis [15,16,17], fatty acid profiles [18,19,20], and optical spectroscopy techniques [21,22,23]. These techniques, alone or combined, produce large datasets that can be analyzed either through classical statistical techniques or by advanced chemometric approaches [14,21,22,24,25].

Considering optical spectroscopy techniques that generate large amounts of data, the use of statistical chemometric approaches for data analysis is especially valuable since these techniques provide a way to visualize variation or patterns within large multivariate data sets and enable the subsequent application of calibration or classification models [21,23,26,27]. The most common optical spectroscopy techniques used for food traceability purposes are vibration spectroscopy [27] and near-infrared or Fourier transform infrared technologies [21,23,26]. These techniques produce spectral information on the food sample, and it is the analysis of this (raw or normalized) optical data through chemometrics that allows for interpretations and identifications of chemical or biochemical compounds independently of highly specialized technicians or chemistry or biochemistry researchers [26].

Total reflection X-ray fluorescence (TXRF) spectroscopy elemental data have a high degree of accuracy in depicting elemental signatures of seafood products of different geographical origins [12,13,14]. Nevertheless, as with other spectroscopy techniques, the generated spectra contain more information than the one used to calculate specific indexes or compound concentrations [21,23,26,28]. Considering optical spectroscopy techniques, the use of statistical chemometric approaches for data analysis is especially valuable because these techniques generate large amounts of data. Thus, the analysis of TXRF full spectral data through chemometric approaches can improve the capability of this analytical technology for geographical traceability purposes, improving the classification accuracy of seafood provenance beyond element-concentration-based classifications [28].

In the present work, we aimed to evaluate the applicability of TXRF full spectral data coupled with chemometric models to depict the geographical provenance of muscle tissue samples of five economically relevant marine species harvested in different areas of the Portuguese Northeast Atlantic coast.

## 2. Materials and Methods

### 2.1. Sample Collection

**Figure 1:**
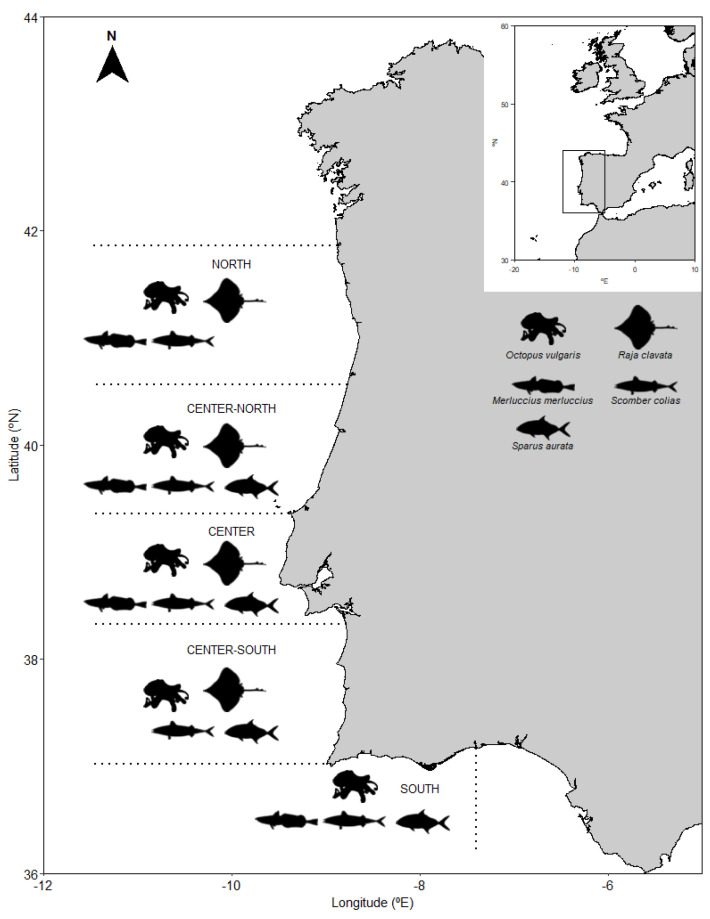
*Merluccius merluccius*, *Octopus vulgaris*, *Raja clavata*, *Sparus aurata*, and *Scomber colias* sampling sites along the Portuguese Atlantic coast.

Five species of seafood were collected from commercial fisheries in five fishing areas along the North Atlantic Portuguese coast (Figure 1), namely European hake (*Merluccius merluccius*), Atlantic chub mackerel (*Scomber colias*), gilthead seabream (*Sparus aurata*), thornback ray (*Raja clavata*), and common octopus (*Octopus vulgaris*). A total of 649 individuals were collected, with four out of five areas sampled per species (except for *O. vulgaris* and *S. colias,* which were sampled in all five areas), and with 30 individuals (i.e., replicates) per species per area (except for the center-south area where only 19 individuals of *R. clavata* were sampled). Individuals were transported fresh to the laboratory, where they were individually dissected to collect muscle tissue samples for elemental analysis and subsequently stored at −80 °C and then freeze-dried before chemical analysis.

### 2.2. Sample Processing and TXRF Analysis

All labware used for TXRF analysis was decontaminated in acid baths for 48 h before use. Freeze-dried samples (approximately 200 mg) were mineralized with HNO_3_ in Teflon reactors, following a microwave digestion process (Multiwave GO, Anton Paar GmbH., Graz, Austria) according to the EPA 3052 method [29]. After cooling, an internal standard (gallium) was added to each sample, and 5 μL of each sample was then applied to a siliconized quartz disk (BrukerNano, Berlin, Germany) and dried. Total reflection X-ray fluorescence spectroscopy was performed in a TXRF S2 PICOFOX (Bruker, Germany). Instrumental recalibration (gain correction, sensitivity analysis, and multi-elemental standards) and analytical blanks were used for quality control. The data were acquired using Spectra PICOFOX Software (version 7.8.20. Bruker, Berlin, Germany).

### 2.3. Spectrum Data Processing and Chemometric Analysis

Specific transformations are commonly applied before the application of partial least-squares discriminant analysis, aiming to reduce the unwanted effects of light scatter caused by the intrinsic physical structure features of the medium of the sample [30]. Among the most common transformations performed on spectral data are the first and second derivatives, allowing for the removal of vertical offsets and linearly sloping baselines [30]. One of the most common algorithms used for this purpose is the Savitzky–Golay transformation [31]. This transformation is based on a localized linear regression of several neighboring points to determine the appropriate polynomial. This polynomial can be mathematically differentiated and evaluated at the x values (in this case, energy values) and, in practical terms, is a mathematical equivalent of the regression. The differentiation procedure is performed by a convolution with a set of derived coefficients [32].

A Savitzky-Golay first-order smoothing normalization coupled with the first derivative of the spectra data was performed using the *mdatools* package [33]. A size window of three points was used throughout the whole spectral range, having as a basis the raw spectral data obtained from the sample analysis. Savitzky-Golay parameters were selected according to the literature, allowing for a more direct comparison of the results obtained with previously reported results. Savitzky-Golay processing of spectral data was performed for all the biological replicates. After pre-processing, the datasets consisted of 649 individuals (generally 30 replicate individuals per sampling site * species; 4 to 5 sites per species, as described in Section 2.1) with 3025 variables in each spectral dataset.

For the chemometric approach, a partial least-squares discriminant analysis (PLS-DA) methodology was used, and a variable selection method was implemented, specifically variable importance in projection (VIP) of PLS-DA. Both analyses were performed using the *DiscriMiner* package [34] in R-Studio Version 1.4.1717 [35]. Cross-validation was performed using the leave-one-out function of the package, and the percentage of correct classification to the known geographical origin of the sample in cross-validation was used as a measure of model accuracy. For the leave-one-out cross-validation procedure and considering the N classes considered for each species, for each ith case in (1, 2, …, N), the data were tested (except for the ith case) to build the classifier model. After this procedure, the model was applied to the ith case, and its classification was evaluated. This procedure was repeated N times, allowing all cases to be assigned to a classification label and the model accuracy evaluated. According to previous works [36], leave-one-out cross-validation is the most adequate for small sample size studies in comparison with resubstitution and simple split-sample estimates that lead to serious bias, with the leave-one-out cross-validation being the method with the smallest bias for discriminant analysis. For each species, the number of components for the model was set as k − 1, where k is the number of geographical origins of the species. The model performance was evaluated using the receiver operating characteristic (ROC) area under the curve (AUC) parameter, the goodness of fit or explained variation (R^2^), and the goodness of prediction or predicted variation (Q^2^). ROC is a probability curve of the false positive rate in the x-axis (i.e., FPR = 1—specificity, where specificity = true negative/(true negative + false positive); or FPR = false positive/(true negative + false positive)) versus true positive rate or sensitivity in the y-axis (i.e., TPR or sensitivity = true positive/(true positive + false negative)). AUC represents the proportion of cases when the model can distinguish between classes. In the present case, the model assigned a sample to one of several possible geographical origins, for example, for *M. merluccius* from the north: true positives (samples from the north are assigned to the north), false positive (samples not from the north are assigned to the north), true negative (samples not from the north not assigned to the north), and false negative (samples from the north not assigned to the north).

The statistical significance of the AUC parameter was evaluated using a Wilcox test. Component selection using ROC-AUC was performed using the *MixOmics* package [37]. After ensuring the correct number of components and high AUC values, model accuracy variable components coordinates were calculated using the *DiscriMiner* package [34].

The two parameters (R^2^ and Q^2^: goodness of fit or explained variation (R^2^) and goodness of prediction or predicted variation (Q^2^), respectively) differently vary with increasing model complexity. The parameter R^2^ is inflationary and approaches 1 as model complexity (number of model parameters) increases. Therefore, it is not sufficient to only consider a high R^2^. The parameter Q^2^, on the other hand, is not inflationary and, at a certain degree of complexity, will not improve any further and will then degrade. Models’ performances in internal validation were evaluated in terms of accuracy (%), sensitivity (%), and specificity (%), according to [38]. The model’s overall accuracy was calculated by dividing the number of correctly classified samples by the total number of samples.

## 3. Results and Discussion

**Figure 2:**
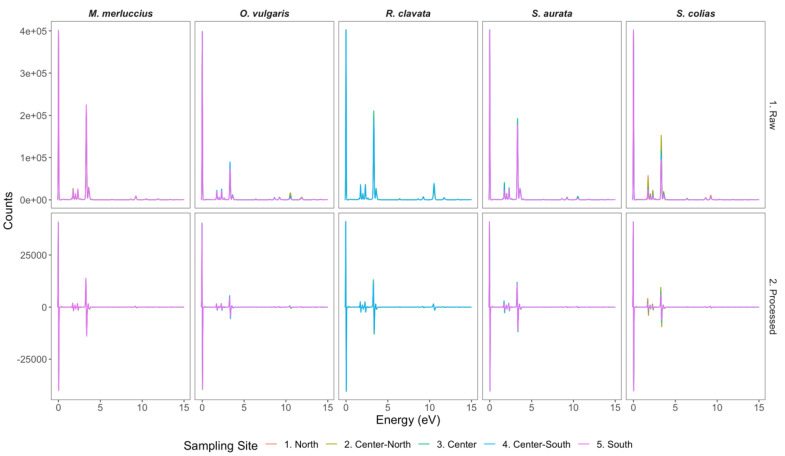
Average raw (upper panel) and processed (lower panel; Savitzky–Golay filter with the first-order differentiation) X-ray fluorescence reflectance spectra of the *Merluccius merluccius*, *Octopus vulgaris*, *Raja clavata*, *Sparus aurata*, and *Scomber colias* samples collected in 5 areas along the Portuguese coast (N = 30 per site per species, except for *R. clavata* in center-south area).

Applying a first-order differentiation Savitzky–Golay transformation to the TXRF spectra allowed for normalization of all samples collected from different organisms, reducing the potential matrix physical effects on the light scattering of the X-ray beam while maintaining the spectral differences inherent to the chemical composition of the samples (Figure 2). The Savitzky–Golay transformation also allowed the removal of baseline effects, mainly but not entirely due to the derivative, and reduced scaling variations [39]. This normalization step was essential to remove the physical light-scattering effects of the matrix, which would have had overfitting effects on the subsequent PLS-DA of the spectra dataset.

**Figure 3:**
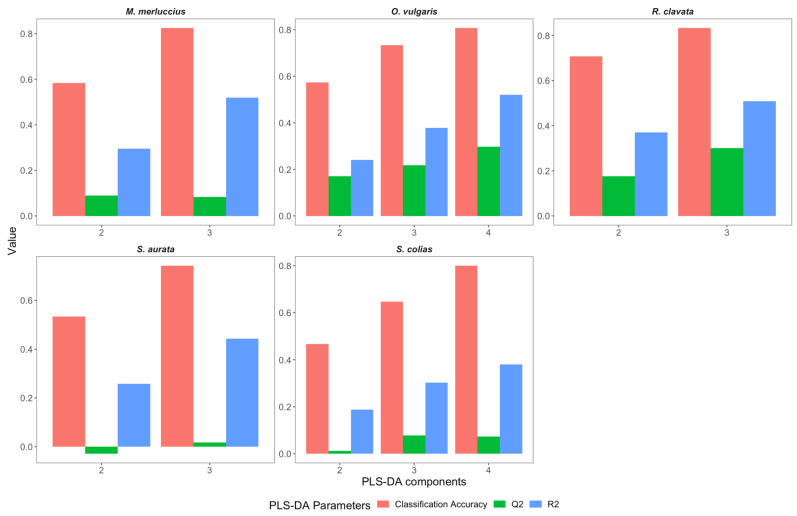
Overall cross-validation classification accuracy (accuracy), goodness of fit or explained variation (R^2^), and goodness of prediction or predicted variation (Q^2^) of the PLS-DA models, having as input the processed (Savitzky–Golay filter with the first derivative) X-ray fluorescence reflectance spectra for each number of components tested for the five studied species *Merluccius merluccius* (4 sites), *Octopus vulgaris* (5 sites), *Raja clavata* (4 sites), *Sparus aurata* (4 sites), and, *Scomber colias* (5 sites,) samples collected along the Portuguese coast. Results for the number of components above k − 1 are not shown.

**Figure 4:**
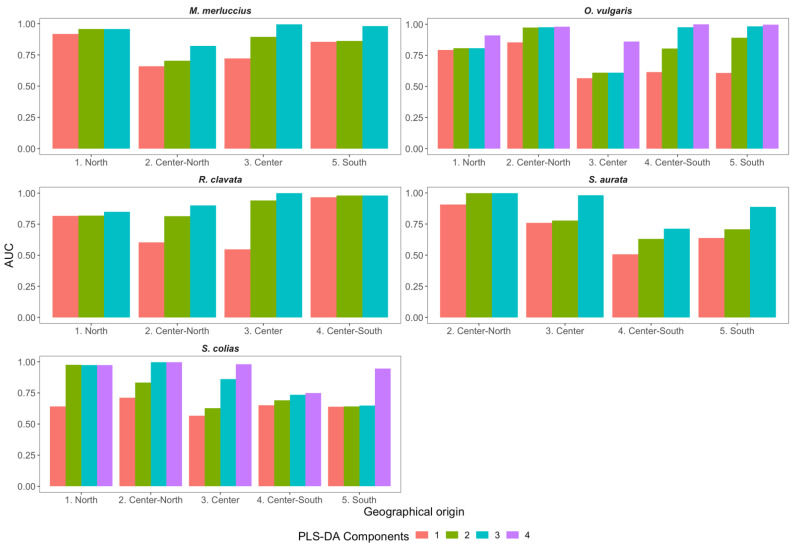
Area under the curve (AUC) of the receiver operating characteristic (ROC) curve of the partial least-squares discriminant analysis (PLS-DA), having as input the processed (Savitzky–Golay first-order differentiation filter) X-ray fluorescence reflectance spectra, conducted for each species (*Merluccius merluccius*, *Octopus vulgaris*, *Raja clavata*, *Sparus aurata,* and *Scomber colias*) and sample geographical origin and number of components. Results for the number of components above k − 1 are not shown.

To select the best PLS-DA components, an evaluation of R^2^ and Q^2^ (Figure 3), as well as of AUC (Figure 4), was obtained from the ROC curves (Supplementary Figure S1). Moreover, the model classification accuracy was assessed. The best model was selected when both Q^2^ and R^2^ were maximized while maintaining an overall high classification accuracy of the model. In this sense, a number of components of k − 1 were selected (where k is the number of geographical origins of the species) as the best model (i.e., the model with the best combination of R^2^, Q^2,^ and accuracy) (Figure 3). In contrast, Q^2^ decreased when the number of components was above k − 1, despite continuing increases in R^2^ and classification accuracy (results not shown).

Additionally, the R^2^, Q^2^, and classification accuracy for each species and the number of PLS-DA components were also compared with the ROC-AUC (Figure 4 and Supplementary Figure S1). This comparison further supported that the best choice of components was k − 1 (where k is the number of geographical origins of each species), for which AUC (Figure 4) and sensitivity (Supplementary Figure S1) were the highest.

**Figure 5:**
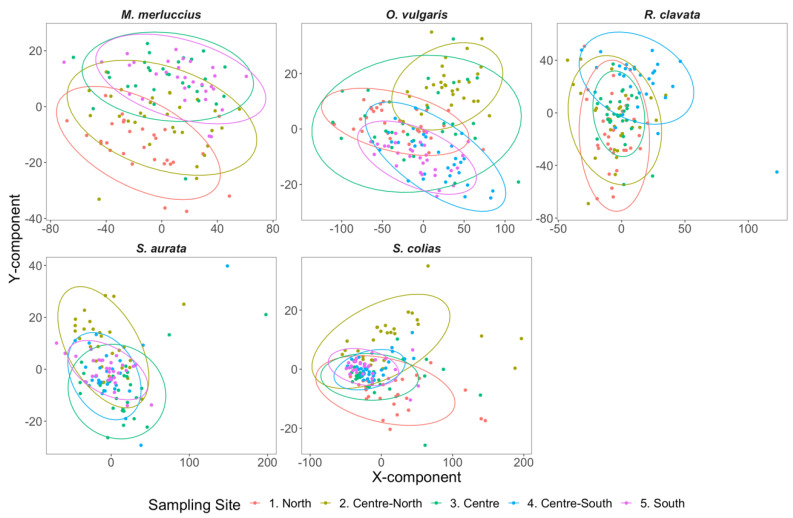
Partial least-squares discriminant analysis (PLS-DA) 3D plots of the first three PLS-DA components having as input the processed (Savitzky–Golay first-order differentiation filter) X-ray fluorescence reflectance spectra of *Merluccius merluccius*, *Octopus vulgaris*, *Raja clavata*, *Sparus aurata,* and *Scomber colias* samples collected along the Portuguese coast (average N = 30 per site per species).

**Figure 6:**
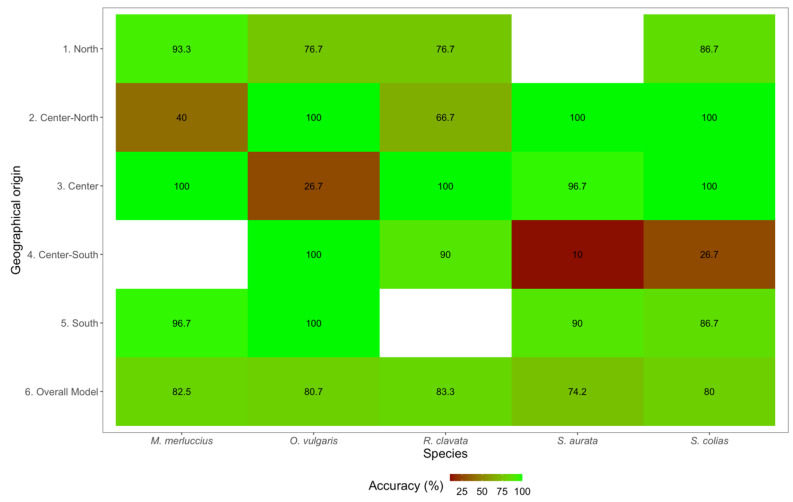
Partial least-squares discriminant analysis (PLS-DA) cross-validation classification accuracy heatmaps per sampling area and for all the different species considered (*Merluccius merluccius*, *Octopus vulgaris*, *Raja clavata*, *Sparus aurata,* and *Scomber colias*).

Following the definition and validation of the number of components per species, PLS-DA 2D plots were generated for visualization of model dispersion, displaying the samples along with the first three components of the PLS-DA (Figure 5). Notably, the generated biplots only represent the data dispersion and grouping in the generated PLS-DA models considering the first two dimensions, whereas the generated models were all obtained using more than two components. This resulted in an apparent complete overlap between some groups, an artifact from the first two components that did not occur when considering all the dimensions used in each model. Several clusters were evidenced in each species dispersion plot, generally grouping samples from the same collection site. In agreement with this definition of clusters, the classification accuracy of models (i.e., percentage of correct classification to the known geographical origin of the samples) was generally high (Figure 6). An overall model classification accuracy above 80.0% was observed for most species, with the exception of the model generated for *S. aurata,* which had a lower overall classification accuracy (74.2%). Previous works [40] also indicated that classifiers attained in PLS-DA approaches can guarantee highly efficient classification results in cross-validation. This might be due to the existence of a direct linear relationship between TXRF spectral patterns and the geographical origin of the considered species, promoted by Savitzky–Golay spectral pre-processing operations, because even in highly complex samples, this correlation can be easily extrapolated by traditional linear techniques [40,41]. For all species analyzed, lower accuracy of classification to geographical origin area (below 75%) was observed in the center areas (i.e., center-north, center, and center-south; Figure 6). This occurred in one area per species and was not limited to a specific type of organism, as the studied marine species have distinct habitat use and biological characteristics: demersal bony fish (*M. merluccius*), pelagic bony fish (*S. colias*), coastal demersal bony fish (*S. aurata*), demersal elasmobranch (*R. clavata*), and benthic cephalopod (*O. vulgaris*). This points to a geographical influence rather than a biological feature, possibly driven by physical-chemical similarities in these coastal areas or by possible capture in areas near the border of adjacent central areas.

In terms of model performance (Table 1), the samples collected in the central part of the study area (center-north, center, and center-south) showed the lowest sensitivity (average sensitivity considering all species of 67.3%). Some of these areas also showed lower precision (average precision of 78.7%), although other areas with high sensitivity presented low precision. This finding is mostly due to the high number of false negatives (samples that were not assigned to their origin), especially evident in *O. vulgaris* and *S. aurata* samples collected in the center area. As for specificity, it was consistently high for all models, except for the *S. aurata* in center-south (74.7%) due to the high number of false positives (samples incorrectly assigned to this origin), thus reducing the specificity of the model for this location.

**Figure 7:**
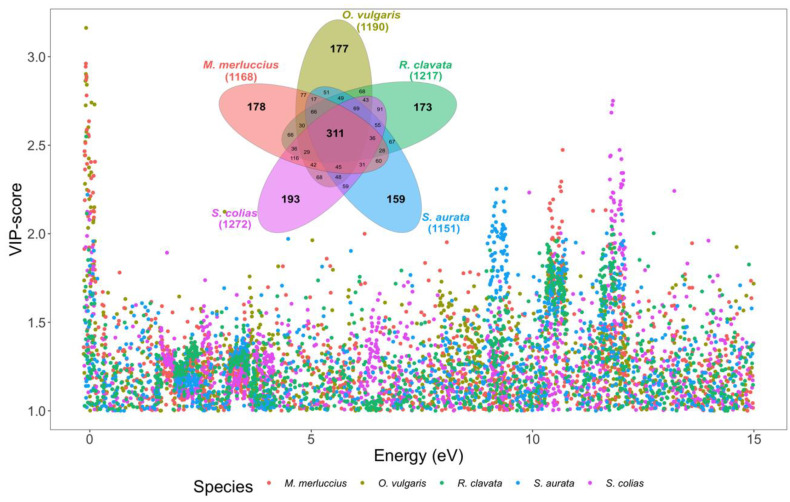
Scatter plot of the VIP scores (only variables with VIP score > 1) attained from the partial least-squares discriminant analysis (PLS-DA) of the processed (Savitzky–Golay first-order differentiation filter) X-ray fluorescence reflectance spectra of the *Merluccius merluccius*, *Octopus vulgaris*, *Raja clavata*, *Sparus aurata,* and *Scomber colias* samples collected along the Portuguese coast, and Venn diagram of the selected variables (VIP score > 1) between the different analysed species.

Most chemometric approaches based on spectral data use near-infrared (NIR) as a basis [21,23,40], where some spectral regions correspond to specific groups of compounds present in the sample matrix (e.g., lipids, carbohydrates, and proteins) [22]. In X-ray fluorescence-based spectral data, peaks result from the excitation of certain chemical elements present in the sample matrix by X-ray photons, generating fluorescence emission peaks, with each element generating two or more tails [28]. Analyzing the spectral features with VIP scores above one from all the models generated for the considered species, it is possible to observe that certain areas of the spectra appear with a higher density of points corresponding to spectral features with VIP > 1 (Figure 7). The Venn diagram revealed that the 311 spectral data points (10% of the spectral features in each dataset) with VIP > 1 were shared among the different species datasets analyzed by the PLS-DA approach, indicating that they are key features for sample class differentiation throughout PLS-DA. On the other hand, the unique features highlighted for each species dataset that were not shared by any other were much more reduced in number, ranging from 159 to 193 (5.3% to 6.4% of the spectral features in each dataset). Although each element can have several fluorescence peaks, certain spectral windows can be associated with groups of elements, whereas for elemental concentration calculation, two or more peaks are normally used for deconvolution. Observing the higher data point density regions, it is possible to distinguish four main peaks with a particularly high density of VIP scores with noticeable high values (VIP > 1.5–2). The first observable peak area corresponds to the beginning of the spectra, where it corresponds to low-atomic-number (Z) elements such as Na, K, and Ca, highly abundant in marine species [12,13,14,42,43]. The last three observable peaks correspond to an area where Cu, Zn, Br, Sr, Pb, and other high-Z elements, have one of the main fluorescence peaks, with these elements also being very abundant in marine seafood samples [12,13,14,42,43]. Nevertheless, the use of specific elements instead of the full TXRF spectra greatly reduces the number of features used as input for the chemometric approaches, from several thousand to a few dozen, as it is observable between elemental analysis and other spectral fingerprinting approaches [40,43]. While for food safety and nutrition analysis, elemental concentration in edible seafood tissues is essential, for provenance and traceability, we can used the full power of the spectral analysis to amplify discrimination and classification success. Nonetheless, XRF spectral data were already previously included in chemometric approaches in several areas from geochemistry to ecology, archaeology, agriculture, material, forensic sciences and medicine [28]. To the best of our knowledge, this is the first time this approach has been employed for food traceability purposes.

## 4. Conclusions

Total X-ray fluorescence (TXRF) analysis provides important elemental data that can be used for food safety and nutritional purposes but also provides a valuable source of spectral data that can be leveraged to boost traceability and provenance applications. Similar to the approach used for infrared spectroscopy, using Savitzky–Golay smoothing normalization coupled with the first derivative approach, it is possible to produce TXRF spectra with significant noise reduction while maintaining discriminant features. Applying PLS-DA to these smoothed spectral datasets was found to be a highly efficient approach to discriminate samples from each species from different sampling areas, with minimum overall model accuracies of 74.2% and individual geographical origins identified with 100% accuracy. It should be emphasized that this approach was achieved for seafood species with very different sample matrixes (muscle tissue from bony and cartilaginous fishes to cephalopods) and habitat use (demersal, pelagic, benthic, and coastal), highlighting the broad applicability of the present methodology. The present methodology is proposed for provenance and traceability purposes. If coupled with classic TXRF spectral peak deconvolution and elemental quantification, it additionally allows for the characterization of different samples in terms of their elemental profiles, providing a highly accurate and informative dataset in terms of food safety.

## Figures and Tables

**Table 1:** Partial least-squares discriminant analysis (PLS-DA) model cross-validation performance (precision, sensitivity, and specificity) per geographical origin area and overall, based on the processed (Savitzky–Golay first-order differentiation filter) X-ray fluorescence reflectance spectra of the *Merluccius merluccius*, *Octopus vulgaris*, *Raja clavata*, *Sparus aurata,* and *Scomber colias* samples collected along the Portuguese coast.

Species	Geographical Origin	Group Precision	Overall Precision	Group Sensitivity	Overall Sensitivity	Group Specificity	Overall Specificity
*M. merluccius*	North	70.0%	82.5%	93.3%	82.5%	85.5%	82.5%
	Center-North	92.3%		40.0%		98.9%	
	Center	93.8%		100.0%		97.2%	
	Center-South	-		-		-	
	South	82.9%		96.7%		92.1%	
*O. vulgaris*	North	59.0%	80.7%	76.7%	80.7%	86.0%	80.7%
	Center-North	83.3%		100.0%		93.8%	
	Center	53.3%		26.7%		94.2%	
	Center-South	100.0%		100.0%		100.0%	
	South	100.0%		100.0%		100.0%	
*R. clavata*	North	76.7%	83.3%	76.7%	83.3%	91.7%	83.3%
	Center-North	71.4%		66.7%		90.9%	
	Center	100.0%		100.0%		100.0%	
	Center-South	84.4%		90.0%		93.6%	
	South	-		-		-	
*S. aurata*	North	89.7%	73.3%	100.0%	73.3%	96.7%	73.3%
	Center-North	88.2%		96.7%		89.4%	
	Center	73.2%		10.0%		97.7%	
	Center-South	88.9%		86.7%		74.7%	
	South	-		-		-	
*S. colias*	North	89.7%	80.0%	86.7%	80.0%	96.9%	80.0%
	Center-North	88.2%		100.0%		95.7%	
	Center	73.2%		100.0%		89.1%	
	Center-South	88.9%		26.7%		99.1%	
	South	70.3%		86.7%		89.5%	

## Data Availability

Data available upon reasonable request.

## Supplementary Materials

The following supporting information can be downloaded at: https://www.mdpi.com/article/10.3390/foods11172699/s1, Figure S1: Receiver operating characteristic (ROC) curves for the number of selected partial least-squares discriminant analysis (PLS-DA) components having as input the processed (Savitzky–Golay filter with the first derivative) X-ray fluorescence reflectance spectra of *Merluccius merluccius*, *Octopus vulgaris*, *Raja clavata, Sparus aurata*, and *Scomber colias* samples collected along the Portuguese coast.
