# Supplementary material for: Harnessing the Full Power of Chemometric-Based Analysis of Total Reflection X-ray Fluorescence Spectral Data to Boost the Identification of Seafood Provenance and Fishing Areas
Source: Foods. 2022 Sep 4;11(17):2699. doi: 10.3390/foods11172699 (PMC9455438; doi:10.3390/foods11172699)
Supplement: Supplementary file 1 [file foods-11-02699-s001.zip › foods-1883132-supplementary.pdf]

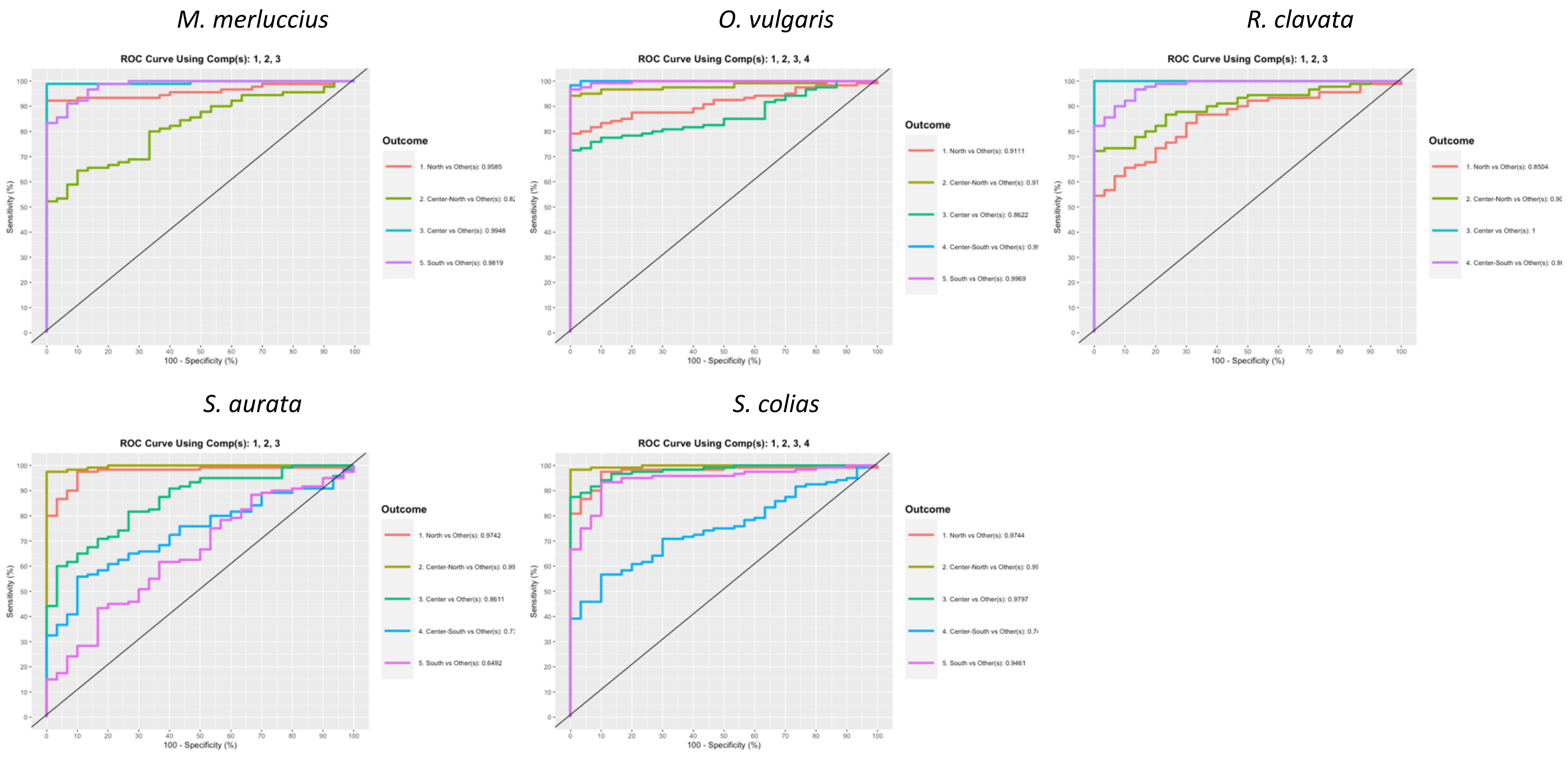

Figure S1. Receiver Operating Characteristic (ROC) curves for the number of selected Partial Least-Squares Discriminant Analysis (PLS-DA) components having as input the processed (Savitzky-Golay filter with the first derivative) X-ray fluorescence reflectance spectra of *Merluccius merluccius*, *Octopus vulgaris*, *Raja clavata, Sparus aurata* and *Scomber colias* samples collected along the Portuguese coast.
